# Clinical and Histological Assessment of Knife-Edge Thread Implant Stability After Ridge Preservation Using Hydroxyapatite and Sugar Cross-Linked Collagen: Preliminary Report

**DOI:** 10.3390/dj13120585

**Published:** 2025-12-08

**Authors:** Lidija Veljkovic, Miljana Nedeljkovic, Gvozden Rosic, Dragica Selakovic, Nemanja Jovicic, Momir Stevanovic, Jovana Milanovic, Aleksandra Arnaut, Milica Vasiljevic, Pavle Milanovic

**Affiliations:** 1 Department of Dentistry, Faculty of Medical Sciences, University of Kragujevac, 34000 Kragujevac, Serbia; veljkoviclidija008@gmail.com (L.V.); miljananedeljkovic999@gmail.com (M.N.); momirstevanovic7@gmail.com (M.S.); jovannakg94@gmail.com (J.M.); sandra11_92@yahoo.com (A.A.); milicavaska13@gmail.com (M.V.); 2 Department of Physiology, Faculty of Medical Sciences, University of Kragujevac, 34000 Kragujevac, Serbia; grosic@fmn.kg.ac.rs (G.R.); dragica984@gmail.com (D.S.); 3 Department of Histology and Embryology, Faculty of Medical Sciences, University of Kragujevac, 34000 Kragujevac, Serbia; nemanjajovicic.kg@gmail.com

**Keywords:** alveolar ridge preservation, bone regeneration, implant design, knife-edge thread, implant stability

## Abstract

**Background**: Primary stability of dental implants depends on bone quality, bone quantity, and implant design. In cases of large defects, such as periapical lesions, the selection of an appropriate alveolar ridge preservation (ARP) material is crucial for bone regeneration and preparation for implant placement. Objective: The aim of this study was to evaluate clinical and histological outcomes of a novel ARP material hydroxyapatite and sugar cross-linked collagen (HSCC) combined with a knife-edge thread implant (KTI) design. **Methods**: Thirty patients were divided into two groups: a control group treated with KTI after spontaneous alveolar ridge healing, and an experimental group that underwent ARP using HSCC, and six months later, KTIs were placed in newly formed bone. Clinical parameters including insertion torque value (ITV), resonance frequency analysis (RFA), implant stability quotient (ISQ), and horizontal bone dimension were evaluated. Histological analysis was also performed. **Results**: No significant differences were observed between groups in ITV, ISQ, or horizontal bone dimension (*p* > 0.05). However, histological analysis demonstrated a significantly higher number of active osteoblasts in the ARP group compared to the control (*p* < 0.001), whereas collagen deposition was significantly greater in the control group (*p* < 0.001). **Conclusions**: ARP using HSCC, combined with KTI, provides favorable conditions for primary stability and successful graft integration, supporting reliable implant placement in sites with bone defects.

## 1. Introduction

One of the most frequently performed interventions in oral surgery is tooth extraction [1,2]. Tooth extraction leads not only to esthetic challenges, but also to significant functional impairments in daily activities [3]. Following extraction, therapeutic strategies may differ [4]. The conventional approach relies on natural healing and spontaneous alveolar ridge regeneration over time [5]. In contrast, the concept of modern and regenerative dentistry emphasizes the replacement of missing teeth within a short period after extraction or the use of alveolar ridge preservation (ARP) techniques to achieve optimal conditions for implant placement [6,7]. Adjunctive therapies such as ozone and hyaluronic acid have shown potential to modulate inflammation and support early tissue healing, which is particularly important from the perspective of regenerative dentistry [8].

To achieve favorable conditions for implant placement, particularly in cases of extensive periapical lesions, implantologists recommend ARP, which is now considered the gold standard in contemporary implant dentistry [9]. A variety of ARP techniques have been proposed, such as conventional approaches, the use of allografts, xenografts, and barrier membranes [10]. On the other hand, novel ARP techniques include PRF, PRF combined with bone graft materials, injectable bone repair materials, and bioactive materials [11,12,13,14]. Each technique presents both advantages and disadvantages; however, their common objective is the preservation of the alveolar ridge volume and quality, thereby facilitating successful implant placement [15]. When selecting the most appropriate technique, clinicians must consider multiple factors, including minimally invasive protocols, cost-effectiveness, predictability, safety, and high clinical success rates [9,16]. One of the novel techniques is ARP using Hydroxyapatite and Sugar Cross-Linked Collagen (HSCC) (Ossix^®^ Bone, Datum Dental, Lod, Israel). Abundo and coworkers [17] listed numerous advantages of HSCC, such as the lack of need for flap elevation and the addition of a membrane barrier to cover the graft, reducing invasiveness, complexity, and costs of the treatment.

Also, understanding the biological behavior of bone grafting materials requires rigorous histological evaluation. Ashman and collaborators [18] were among the first to perform histological and clinical studies on ARP sites using a calcified, microporous copolymer graft material. Even after 25 years, histological analyses remain fundamental for evaluating bone substitutes, with research focusing on the stages of ossification, cellular composition, collagen content, growth factors, and residual graft particles [19,20]. These findings guide clinicians in optimizing bone regeneration procedures and improving clinical outcomes.

In addition to ARP, implant design plays a critical role in the success of implant therapy [21]. Over the years, numerous implant macro- and micro-designs have been introduced, all aiming to enhance osseointegration [22]. Achieving high primary stability remains the cornerstone of implant success, which depends both on bone quality and implant design [23]. Nandini and colleagues [24] demonstrated that implants with a conical body achieve higher primary stability compared to cylindrical implants. Furthermore, knife-edge thread implants (KTI), deep threads and a higher pitch facilitate not only improved primary stability, but also secondary stability [25]. Moreover, the AnyRidge^®^ implant system (Megagen, Daegu, Republic of Korea) has been specifically developed to provide high insertion torque values (ITV), even in bone type D4 [26]. Farronato and collaborators [27] described the role of KTI in improving ITV and primary stability by achieving intimate contact with the cancellous bone, particularly in soft bone. Another advantage of the knife-edge design is its improved interlocking capability, which provides optimized load distribution and enhances biomechanical engagement with the surrounding bone [27,28].

For clinicians, understanding the role of thread geometry is highly valuable. It is known that KTI geometry offers a wider external surface area compared to V-shaped threads [27]. Finite element analyses also show that thread geometry significantly impacts stress distribution, influencing both implant stability and the health of the surrounding bone [29]. Other geometric factors such as thread pitch, depth, and width contribute to increasing bone–implant contact (BIC) as well as optimizing load distribution, thereby supporting long term success [30].

However, ITV is not the only parameter of stability. To further evaluate implant stability immediately after placement, it is strongly recommended to perform resonance frequency analysis (RFA) [31]. RFA is interpreted through implant stability quotient values (ISQ), which range from 0 to 100, with values between 60 and 70 being particularly relevant in clinical practice for assessing primary and secondary stability [26]. Also, ISQ values serve as an important parameter for clinicians when deciding on loading protocols and predicting long-term implant survival [32].

Despite the extensive literature, there are still few studies examining the quality of newly formed bone tissue after ARP as well as the clinical behavior of implants in this tissue. Therefore, the aim of this study was to evaluate histological parameters after ARP using hydroxyapatite combined with sugar cross-linked collagen, as well as to assess clinical parameters of knife-edge thread implants such as ITV and ISQ, following implant placement in the augmented ridge. Accordingly, the tested hypothesis was that HSCC provides an effective ARP modality and that KTIs achieve satisfactory primary and stability (ITV and ISQ) within the newly formed bone, together representing a clinically effective combination.

## 2. Materials and Methods

### 2.1. Study Design and Ethical Considerations

This study was conducted at the Department of Dentistry, Faculty of Medical Sciences, University of Kragujevac, Serbia, between January and August 2025. This research was performed in accordance with the principles of the Declaration of Helsinki and approved by the Institutional Review Board of the Faculty of Medical Sciences, University of Kragujevac (approval ID: 09-666/8). All participants provided written informed consent prior to enrollment.

### 2.2. Patient Selection

A total of 30 patients were included (14 males and 16 females), aged 38 ± 9 years, based on the following inclusion criteria: age ≥ 18 years, presence of periapical lesions in the maxillary first molar region with interradicular septum destruction, good systemic health, and non-smoker status. Exclusion criteria were patients without posterior teeth and those with systemic conditions known to compromise bone regeneration (osteoporosis or diabetes mellitus). The total sample size of 30 implants (15 per group) was determined based on feasibility and methodological consistency with previously published clinical research evaluating implant stability. Notably, Sansupakorn and coworkers [33] conducted a controlled clinical study including 30 implants (15 per group), demonstrating that such a sample size is appropriate for detecting clinically meaningful differences in ISQ values between treatment groups.

### 2.3. Study Groups and Surgical Procedure

Patients were divided into two groups (15 in each group).

1. Control group: patients who had undergone extraction of the maxillary first molar at least eight months earlier, allowing spontaneous alveolar ridge healing and implant placement.
2. Experimental group: patients who had undergone extraction of the maxillary first molar followed by ARP with HSCC and implant placement.

Tooth extractions were performed using a standardized protocol by the same experienced oral surgeon (P.M.). Local infiltration anesthesia (Articaine with 1:100,000 epinephrine) was administered, followed by elevation of a full-thickness mucoperiosteal flap. Root separation was performed in order to facilitate atraumatic removal of the tooth. After extraction, ARP was carried out using HSCC (5 × 10 × 10 mm, Ossix^®^ Bone, Datum Dental, Lod, Israel). Upon completion of the procedure, single interrupted sutures (polypropylene 5.0, Yavo) were placed to protect the graft. Sutures were removed after seven days. Six months after ARP, cone-beam computed tomography (CBCT) scans were obtained, and the horizontal dimensions of the alveolar ridge were evaluated 1 mm from the crestal bone. Based on the available bone volume, appropriate implants (AnyRidge^®^ implant system, Megagen, Daegu, Republic of Korea) were selected for placement.

Second surgery included punch biopsy and implant placement. All surgeries were performed by P.M. according to a standardized protocol. Local infiltration anesthesia (Articaine with 1:100,000 epinephrine) was administered followed by full-thickness flap elevation. A trephine bur (5 × 3.5 mm) was initially used to harvest bone samples for histological evaluation. Subsequently, the osteotomy site was prepared with sequential implant burs, and implants were placed using a surgical handpiece at 40 rpm with a torque of 45 Ncm. After implant insertion, both ITV and ISQ measurements were recorded using a resonance frequency analysis device (MegaISQ II, Megagen, Daegu, Republic of Korea). Primary closure was achieved using interrupted sutures (polypropylene 5.0, Yavo). Sutures were removed after seven days.

**Figure 1:**
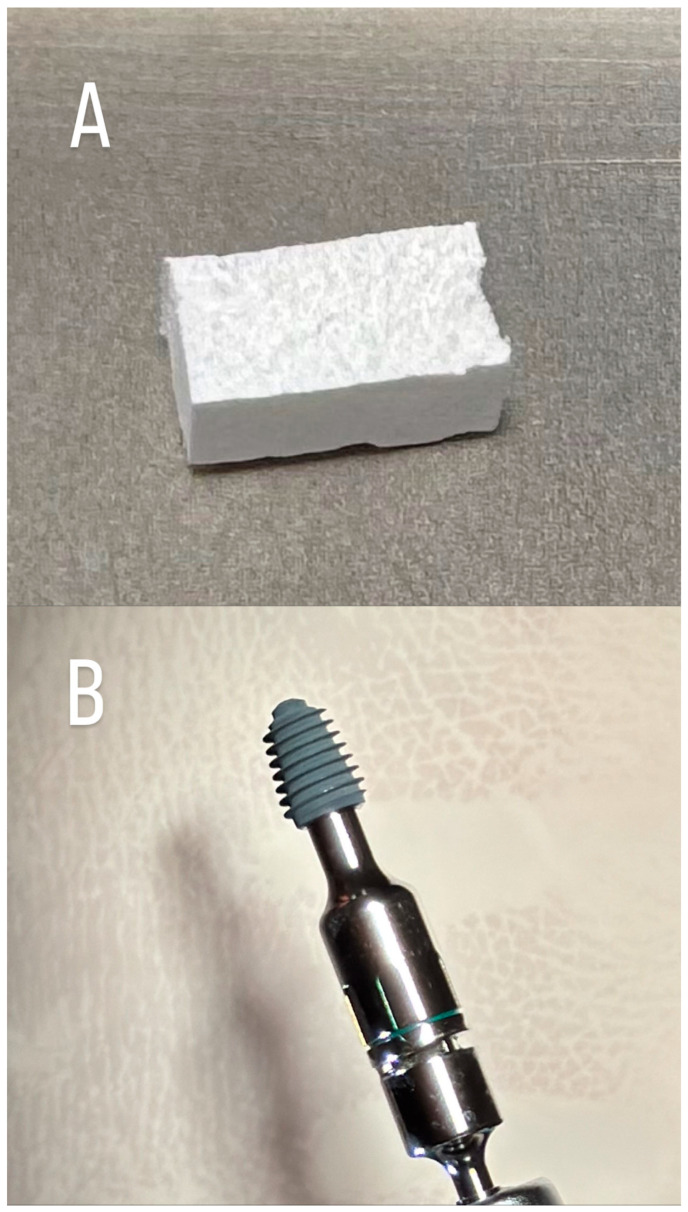
Graft material and dental implant. (**A**) Hydroxyapatite and Sugar Cross-Linked Collagen (HSCC) (Ossix^®^ Bone, Datum Dental, Lod, Israel); (**B**) Knife-Edge Thread implant (KTI) (AnyRidge^®^ implant system, Megagen, Daegu, Republic of Korea).

**Figure 2:**
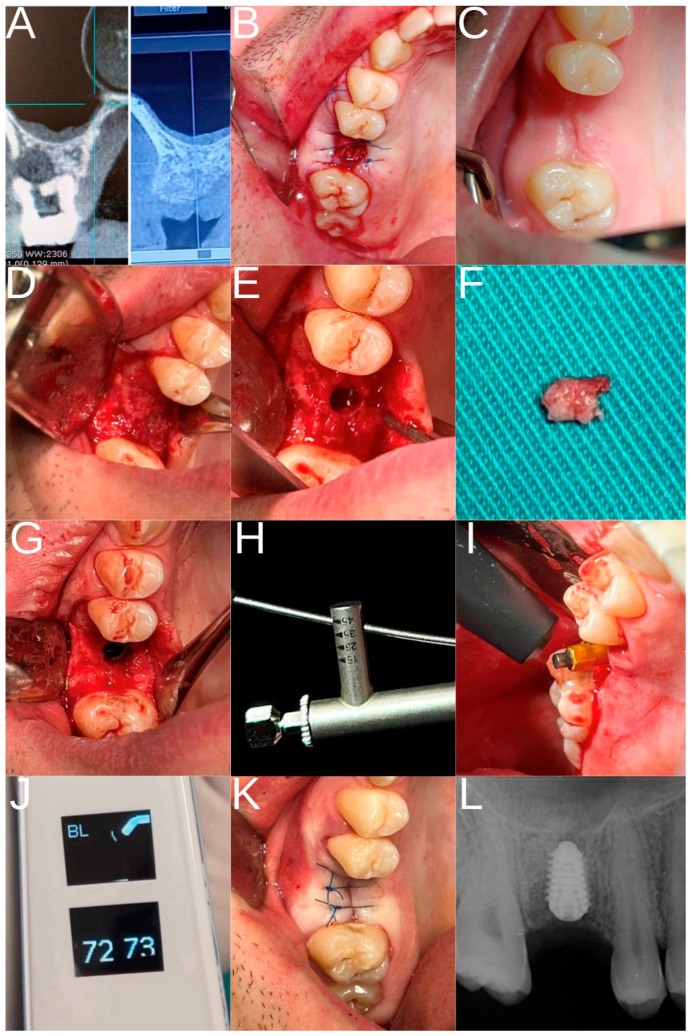
Surgical procedure. (**A**) CBCT scan of initial situation and six months after alveolar ridge preservation (ARP); (**B**) Immediate after tooth extraction and ARP; (**C**) Soft tissue condition after six months; (**D**) Full flap thickness elevation and bone condition; (**E**) Punch biopsy; (**F**) Part of bone for the pathohistological evaluation; (**G**) Implant placement; (**H**) Torque estimation; (**I**) implant stability quotient values ISQ estimation; (**J**) ISQ values; (**K**) Primary closure; (**L**) Periapical radiograph taken after implant placement shows proper position.

HSCC as well as KTI are presented in Figure 1. In addition, Figure 2 shows the surgical procedure.

### 2.4. Postoperative Care

All patients received systemic antibiotic therapy (Amoxicillin 500 mg, three times daily; Hemofarm, Vršac, Serbia), anti-inflammatory medication (Ibuprofen 400 mg, three times daily; Mylan Hungary Kft, Komarom, Hungary), and antiseptic mouth rinses (Peri Plus+ 0.09%, Curadent AG, Kriens, Switzerland) for six days. No provisional restorations were delivered prior to the scheduled implant prosthetic rehabilitation.

### 2.5. Histological Analysis

Following surgical processing, bone samples were fixed in 10% formalin for 24 h. Decalcification was carried out using OsteoFast 2 (Biognost, Zagreb, Croatia) over a period of 12 days, with the solution replaced every three days. After confirming complete decalcification, the tissues were washed in distilled water for 30 min, after which automated tissue processing was initiated. The specimens were dehydrated through a series of graded ethanol concentrations and xylene, followed by embedding in paraffin. Paraffin blocks were sectioned into slices 5–7 µm thick using a microtome and mounted on adhesive slides.

The tissue samples were stained with hematoxylin-eosin and Picrosirius red according to standard protocols. Images were captured using an optical microscope (Leica DM2500, Wetzlar, Germany) fitted with a digital camera. For the quantitative assessment of collagen deposition, bright-field images of Sirius red-stained sections were taken at 20× magnification, and the positive areas were quantified using ImageJ software version 1.54a (National Institutes of Health, Bethesda, MD, USA). Histological scoring and evaluations were performed in a blinded manner by two independent observers. Findings are presented as mean cell count per field or mean percentage of positive area.

### 2.6. Statistical Analysis

Statistical analysis was performed using SPSS version 20.0 (IBM SPSS Statistics, Chicago, IL, USA). Results are expressed as mean ± standard deviation (SD). The Mann–Whitney U test and, where appropriate, the independent-samples Student’s *t*-test were used to assess statistical significance. A *p*-value of <0.05 was considered statistically significant.

## 3. Results

**Figure 3:**
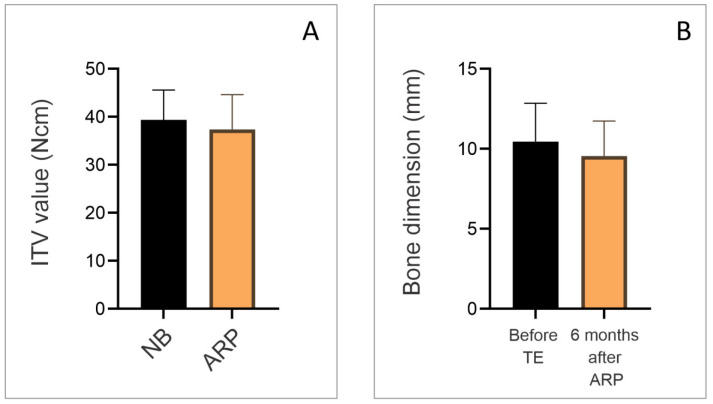
(**A**) Insertion torque value (ITV) during implant placement in two groups: natural bone (NB) and ARP. (**B**) Horizontal bone dimensions: Before tooth extraction and 6 months after alveolar ridge preservation.

The analysis of ITV during implant placement is presented in Figure 3A. The mean ITV was comparable between the two groups: natural bone (NB) and ARP group. In both groups, values were approximately 35–40 Ncm, with no statistically significant differences observed (*p* > 0.05). Figure 3B illustrates changes in horizontal alveolar ridge dimensions. Prior to tooth extraction (TE), the mean ridge width was around 11 mm. Six months after ARP, a slight reduction was observed, with values decreasing to approximately 10 mm. Although this reduction indicates a trend toward dimensional loss, the difference was not statistically significant (*p* > 0.05).

**Figure 4:**
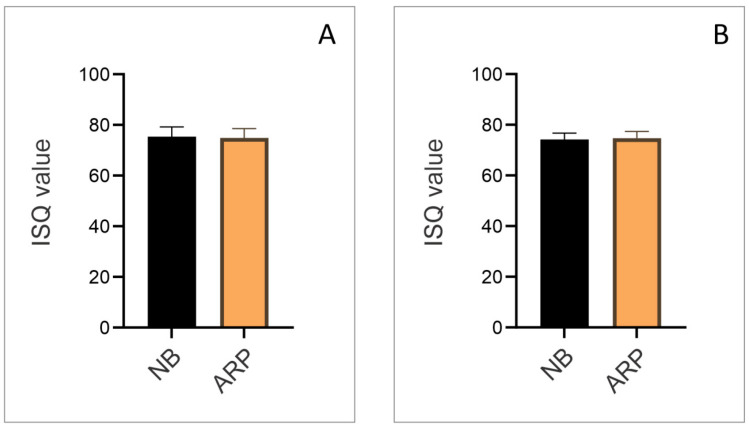
ISQ values immediate after implant placement in two groups: (**A**) ISQ values in buccolingual direction; (**B**) ISQ values in mesiodistal direction.

Figure 4 shows the ISQ values measured immediately after implant placement. In the buccolingual direction (Figure 4A), mean ISQ values were similar between the two groups. The NB group demonstrated a mean ISQ of approximately 74, while the ARP group presented a nearly identical mean value, also around 73–74. No statistically significant differences were detected between the NB and ARP groups (*p* > 0.05). In the mesiodistal direction (Figure 4B), a comparable pattern was observed. Both groups exhibited mean ISQ values close to 75, again with minimal variation and no statistically significant differences (*p* > 0.05).

**Figure 5:**
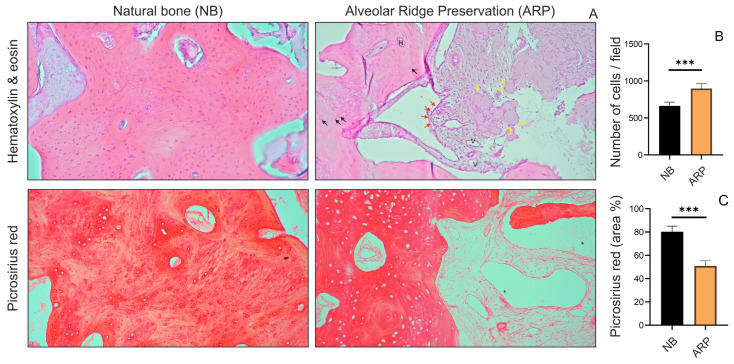
Histological analysis. (**A**) Representative photomicrographs of NB and ARP stained with H&E and Picrosirius red. (**B**) Number of osteoblasts. (**C**) Collagen deposition stained by picrosirius red. (Results are presented as mean percentage of red stained area relative to the total section area ± SEM, *** *p* < 0.001).

Pathohistological analysis of hematoxylin and eosin staining was used to identify and evaluate the cellularity of the investigated regions of the bone tissue. The development of the newly created bone tissue was characterized by the abundance of osteocytes, osteoblasts, blood vessels, and developing Haversian systems. Mature osteocytes were present in the mass of newly formed bone tissue (indicated by the black arrows shown in Figure 5A). The surface of the bone substitute material granules shows erosion where osteoblasts are attached to the material surface (indicated by the yellow arrows shown in Figure 5A). Furthermore, active osteoblasts were positioned along the mineralization front of the newly formed bone (indicated by the red arrows shown in Figure 5A). Obtained results demonstrated that the number of active osteoblasts was significantly higher in the ARP group compared to the natural bone (Figure 5B). To evaluate parameters of new bone formation, we used the selective histochemical technique Picrosirius red. Pathohistological analysis demonstrated that the collagen deposition in the newly created bone was significantly lower in ARP group compared to the natural bone (Figure 5C).

## 4. Discussion

As mentioned earlier, modern implant dentistry aims for immediate implant placement followed by immediate loading, but in cases with insufficient bone caused by large periapical lesions, it is recommended to perform ARP using an appropriate ARP technique. The focus of this research was to evaluate the clinical and histological characteristics of a novel ARP material—HSCC (Ossix^®^ Bone, Datum Dental, Lod, Israel)—and the clinical behavior of implants with a specific thread design (AnyRidge^®^ implant system, Megagen, Republic of Korea) when placed in newly formed, graft-based bone. Previous studies [17,26] reported data related to individual aspects of these analyses (ARP or KTI stability), but to our knowledge, no similar study design combined both clinical and histological examinations to provide a more complete picture.

Our first analysis estimated ITV in two groups of patients. The first condition to allow implant osseointegration is to achieve primary stability. A strong relationship between primary stability and ITV has been confirmed [34]. According to Sarfaraz and coleagues [35], the optimal insertion torque is in the range of 30–60 Ncm. Although our study showed slightly higher ITV in natural bone, there were no statistically significant differences between groups. We did not find another study with the same design for direct comparison, but a similar study was performed by Ko and coworkers [36], who evaluated ITV after ARP performed 10 weeks earlier in the molar site. They reported statistically significant differences between groups, with higher torque values in patients with natural bone (minimum 3 mm ridge width). On the other hand, our lack of significant differences may be explained by the six-month healing period in our study, which allowed regeneration and initial maturation of new bone after ARP. Other factors influencing this parameter include the use of different bone graft materials and implant designs. Numerous studies [26,34] have confirmed that KTIs can achieve good ITV even in D4 bone type. Also, while KTIs are designed to achieve favorable insertion torque values even in more challenging bone conditions, it is important to consider the opposite scenario as well. In cases where implants are placed in D1 type regions, excessively high ITV may become counterproductive. Choosing a thread configuration with a smaller external surface area can help reduce resistance during insertion. When ITV exceeds the physiological tolerance of the bone, localized over-compression may occur, potentially leading to microfractures or ischemic necrosis due to compromised microcirculation [37,38,39,40,41,42,43,44]. From a mechanical point of view, excessively high ITV may also overload the implant abutment interface, resulting in deformation or even fracture. Additionally, excessive resistance during placement may prevent complete subcrestal seating of the implant, leaving a portion of the roughened surface exposed and thereby increasing susceptibility to bacterial contamination and peri-implant inflammatory complications. For these reasons, careful thread design selection and appropriate ITV during surgical planning are essential to maintain adequate primary stability while minimizing biological and mechanical risks.

Beyond primary stability, the dimensional characteristics of the regenerated ridge also represent a critical determinant of implant success. Horizontal bone dimension is the main factor determining implant diameter, and several studies have described the importance of implant diameter for long-term implant success in the maxillary molar area [31,32].

Furthermore, Alquahtani et al. [40] concluded that implant diameter influences micromovements, which may also affect long-term success. Based on this, it is not surprising that we use CBCT to estimate horizontal bone dimensions after ARP. As we analyzed the first maxillary molar region, we evaluated horizontal bone dimensions at a single level (1 mm from the crestal bone). The apical part of the maxillary alveolar ridge usually has sufficient bone, while the crestal part is often the most vulnerable after tooth extraction [34]. In contrast, multiple-level evaluation is recommended for sites such as the central incisor region, where anatomical structures can result in insufficient bone [35]. Although horizontal bone dimensions after ARP were slightly lower compared to pre-extraction, there were no statistically significant differences. In line with our study, Liu et al. [43] also reported no significant differences at 1 mm from the crest after ARP. In contrast, Sun et al. [41] observed significant differences at the same level. These discrepancies may be due to different ARP materials applied across studies. From a clinical standpoint, preserving horizontal bone dimensions is essential for achieving correct three-dimensional implant positioning, which represents a key determinant of long-term implant prosthetic success. Adequate ridge width not only facilitates implant placement but also allows for the selection of an appropriate implant diameter, ensuring sufficient structural strength and stability under functional masticatory loading. Conversely, in cases of inadequate bone volume, various intervention protocols are utilized to establish optimal conditions for implant placement [45,46].

In addition to dimensional bone stability, the functional stability of the implant was assessed through RFA, which provides essential prognostic information. Moreover, the next step in our study was the evaluation of ISQ values. Over the last two decades, ISQ values have become an important tool for both diagnosis and prognosis in implant therapy. As shown in Figure 4, there were no statistically significant differences between fully healed alveolar ridges and those regenerated six months after ARP. In contrast to our results, Vallecillo and colleagues [47] reported mean ISQ values of 75.40 ± 12.80 in natural bone and 67.17 ± 11.47 in regenerated bone. On the other hand, Chen and coworkers [48], in a meta-analysis of randomized controlled trials, reported no statistically significant differences in ISQ between grafted and non-grafted areas. These discrepancies may be explained by variations in grafting materials, membranes, and implant thread designs used across studies. The clinical relevance of the obtained ISQ values is substantial, since their interpretation plays a key role in determining appropriate loading strategies and predicting long-term implant success. Clinically relevant ISQ values are generally considered to fall within the range of 60–70, and numerous studies have reported recommendations on implant stability and loading protocols based on ISQ values [44,49,50,51,52,53,54,55]. Implants with ISQ < 60 are considered to have low stability, and a two-stage implant surgery is highly preferred [52]. For ISQ values between 60 and 64, treatment decisions should depend on the case: in full-arch restorations with splinted implants, immediate loading may be feasible, while single implants should be treated in two stages [51,52]. As ISQ values increase, the “safety zone” for immediate loading broadens. Values of 65–69 represent a safe zone for immediate loading in splinted full-arch cases, while single implants can often be managed with early loading [49,50]. ISQ values ≥ 70 are considered very safe for immediate loading, even for single implants [44,49,55].

Considering the aforementioned values, it is obvious from our results that HSCC provide a suitable substrate for the formation of high-quality bone tissue. Also, KTIs have demonstrated strong stability immediately after placement. Furthermore, mean ISQ values in both buccolingual and mesiodistal directions were ≥70, which, according to clinical protocols, would allow immediate loading. Although immediate loading was not part of our study design, this provides a strong basis for future investigations in this direction.

While mechanical parameters offer valuable insights into implant stability, biological confirmation of graft and new bone stability is equally important. For this reason, a histological evaluation of the regenerated tissue was performed after six months. Histopathological examination shown in Figure 5 revealed a significantly greater degree of cellular activity in the ARP graft samples compared to normal bone tissue. The development of the newly formed bone was marked by an abundance of osteocytes, osteoblasts, and blood vessels. The newly established fibrous bone underwent remodeling into mature cancellous or compact bone, presenting as a lamellar bone structure featuring Haversian systems. While bone regeneration is evident, remnants of the bone substitute material with osteoblasts adhering to its surface were observed in the ARP graft samples. This finding supports the hypothesis that ARP could progressively transform the graft into functional new bone, suggesting the application of HSCC as a bone graft material is promising. Additionally, the histological evaluation of collagen deposition, illustrated in Figure 5, showed that the collagen structures in the ARP group closely resemble the architecture of mature bone. The high concentration of red-stained deposits within ARP samples indicates a dense arrangement of mineralized collagen [56].

These histological findings provide important biological support for the clinical outcomes observed in this study. However, when interpreting the regenerative potential of the tested material, it is also necessary to consider the specific defect characteristics in which it was applied. In the present study, HSCC was evaluated in post-extraction sockets with large periapical lesions in the maxillary first molar region. Although these defects are clinically relevant, they do not necessarily correspond to standardized critical-size defects. Critical size defect models, defined as the smallest bone defects that will not spontaneously and completely heal during the lifetime of the individual, are widely used to test the true regenerative capacity of biomaterials, precisely because spontaneous bone formation is limited in such defects [57]. Ludovichetti et al. [58] demonstrated that in human critical-size alveolar bone defects, deproteinized bovine bone graft significantly improved defect fill compared with spontaneous healing, underscoring the value of challenging defect models when assessing regenerative materials. Current literature does not provide studies on HSCC in critical size defect. Thus, the performance of HSCC in standardized critical-size alveolar defects remains to be clarified, and, as one of the limitations of this study, future studies should specifically address this issue.

Taken together, the consistency between ITV, preserved horizontal bone dimensions, and high ISQ measurements indicates that the regenerated ridge provided mechanical conditions comparable to natural bone. These clinical outcomes were further supported by histological findings demonstrating active remodeling, organized collagen maturation, and progressive integration of the graft material. Such alignment between mechanical stability and biological quality reinforces the suitability of HSCC for ARP and supports the use of KTI in newly formed bone. It should also be noted that the limitations of this study, primarily the small sample size, the absence of longitudinal follow up and the lack of horizontal bone dimension evaluation at different levels, raise important questions for future research aimed at expanding and refining current knowledge. Future investigations should also consider evaluating different intervals of implant placement and histological assessment to better understand HSCC behavior throughout the stages of bone regeneration.

## 5. Conclusions

These findings describe the behavior of a novel ARP material and the clinical parameters of a specific implant design based on knife-edge threads. Based on the study data, it can be concluded that

1. ARP with HSCC enables sufficient bone regeneration in terms of quantity and quality within 6 months;
2. KTIs provide good primary stability and favorable ITV and ISQ values immediately after placement in the ARP area.

## Data Availability

Data will be available upon reasonable request.

## Abbreviations

The following abbreviations are used in this manuscript:

ARP	Alveolar ridge preservation
CBCT	Cone Beam Computer Tomography
ISQ	Implant stability quotient
ITV	Insertion torque value
NB	Natural bone
RFA	Resonance frequency analysis
KTI	Knife-thread implants
HSCC	Hydroxyapatite and sugar cross-linked collagen
